# Hierarchical Multiple Precursors Induced Heterogeneous Structures in Super Austenitic Stainless Steels by Cryogenic Rolling and Annealing

**DOI:** 10.3390/ma16186298

**Published:** 2023-09-20

**Authors:** Duo Tan, Bin Fu, Wei Guan, Yu Li, Yanhui Guo, Liqun Wei, Yi Ding

**Affiliations:** 1 School of Materials Science and Engineering, Shanghai Institute of Technology, Shanghai 201418, China; 2 Baowu Special Metallurgy Co., Ltd., Shanghai 200940, China

**Keywords:** super austenitic stainless steel, cryogenic rolling, heterogeneous structure, microstructure, mechanical properties

## Abstract

Multiple deformed substructures including dislocation cells, nanotwins (NTs) and martensite were introduced in super austenitic stainless steels (SASSs) by cryogenic rolling (Cryo-R, 77 K/22.1 mJ·m^−2^). With the reduction increasing, a low stacking fault energy (SFE) and increased flow stress led to the activation of secondary slip and the occurrence of NTs and martensite nano-laths, while only dislocation tangles were observed under a heavy reduction by cold-rolling (Cold-R, 293 K/49.2 mJ·m^−2^). The multiple precursors not only possess variable deformation stored energy, but also experience competition between recrystallization and reverse transformation during subsequent annealing, thus contributing to the formation of a heterogeneous structure (HS). The HS, which consists of bimodal-grained austenite and retained martensite simultaneously, showed a higher yield strength (~1032 MPa) and a larger tensile elongation (~9.1%) than the annealed coarse-grained Cold-R sample. The superior strength–ductility and strain hardening originate from the synergistic effects of grain refinement, dislocation and hetero-deformation-induced hardening.

## 1. Introduction

Super austenitic stainless steels (SASSs) have been widely used in extremely harsh service environments such as flue gas desulfurization, seawater desalination, and the petrochemical industry [1,2]. However, the yield strength (YS) of SASS is relatively low due to its large grain size and low dislocation density, which strongly limit its further applications. Microstructure refinement is an effective method to improve its YS, and some severe plastic deformation (SPD) methods such as dynamic plastic deformation (DPD) [3,4] and surface mechanical grinding treatment (SMGT) [5,6] are often conducted to obtain the nanostructure; however, they are always accompanied by a significant deterioration in ductility. On this basis, the strategy of heterogeneous structures (HSs), such as bimodal, harmonic and hierarchical lamellar structures, was proposed to achieve a good combination of strength and ductility [7,8,9]. Generally, the commonly available approach to obtain bulk HS in conventional austenitic stainless steels (ASSs) is through annealing after severe cold deformation [10]. The heterogeneity was obtained due to a competitive process between the reverse transformation of strain-induced martensite and the recrystallization of a dislocation-cell-type structure [3], thus resulting in a hierarchical distribution of grain sizes, from tens of manometers to several micro-meters. However, the deformation mode of the studied SASS at an ambient temperature is mainly dislocation-dominated shear band, owing to a heavy alloying of Mo and N and increased stacking fault energy (SFE) [11,12] during cold deformation. The homogeneous rolled substructure hardly contributes to the HS formation due to a similar deformation stored energy for the nucleation and growth of recrystallization [13]. Additionally, dislocation annihilation during recovery or recrystallization impedes structural refinement by walls or cells [14], resulting in low YS.

Recently, an innovative cryogenic rolling (Cryo-R) procedure was performed in conventional ASSs to obtain nanostructures with high YS [15]. An effective inhibition of dislocation recovery during Cryo-R contributed to a larger stored energy and higher driving force for the nucleation of recrystallization compared to the traditional Cold-R [16]. However, most investigations have ignored the advantage of Cryo-R in activating multiple deformed substructures, including dislocation tangles, mechanical twins and deformation-induced martensite (DIM) [12,17], due to a temperature-controlled variable SFE, since the diversity of precursor is a crucial factor in obtaining a heterogeneous grain-size distribution. Firstly, the recrystallization dynamics are closely related to the nucleation site, such as the dislocation cell and twin boundaries [18,19,20]. Some studies have shown that the nanocrystalline nucleation rate in the grain boundary, nanotwin and shear band regions of precursors was faster than that in the dislocation region of coarse crystals during annealing [21,22]. Moreover, the reversed austenite grains may inherit the lath morphology of the DIM after the reverse transformation [23] compared to the equiaxed recrystallized grains. However, little attention has been paid to the effects of deformed precursors with complex substructures in obtaining HS. The motivation of the present study is to investigate the relationship among the HSs, deformation modes and the evolution of multiple precursors during Cryo-R, which provides a guiding design of high-strength SASS for extreme applications.

## 2. Materials and Methods

In this work, the chemical composition of SASS was listed as follows: 0.024 C, 0.38 Si, 0.98 Mn, 0.027 P, 0.004 S, 16.9 Cr, 13.23 Ni, 5.3 Mo, 0.13 N and balanced Fe, wt. %. The cast ingots were cut into 5 × 30 × 100 mm samples and then heated in a muffle furnace at 1200 °C for 2 h for the solution treatment, followed by water cooling. A multi-pass Cryo-R procedure was conducted in a rolling mill (⌀180 mm) with different total reductions of 30%, 50%, and 70%. Before Cryo-R, the iron oxide scale on the surface was removed with coarse sandpaper, and a soak for 15 min was made at a cryogenic temperature. For comparison, Cold-R samples with a 70% reduction according to the same rolling parameters were prepared. Moreover, the 70% rolled samples were subsequently annealed at 700 °C for 10 min. The X-ray (XRD, D/max 2200 PC; Shimadzu, Kyoto, Japan) analysis was conducted by Cu target Kα rays with a scan rate of 2°/min from 40° to 100°. The Vickers hardness values were performed on a 402SXV hardness tester for 5 s with 300 g of load retention. For transmission electron microscope (TEM) observation, the samples were thinned by double-spray electrolysis, and the double-spray liquid was a perchloric acid alcohol solution (voltage and temperature were 30 V and −30 °C, respectively). Then, the samples were observed by FEI TECNAI TEM (FEI, Lexington, KY, USA). The electron backscatter diffraction (EBSD) characterizations of the RD-ND surface were investigated by MIR3 high-resolution scanning electron microscopy at a 20 kV acceleration voltage.

## 3. Results and Discussion

**Figure 1:**
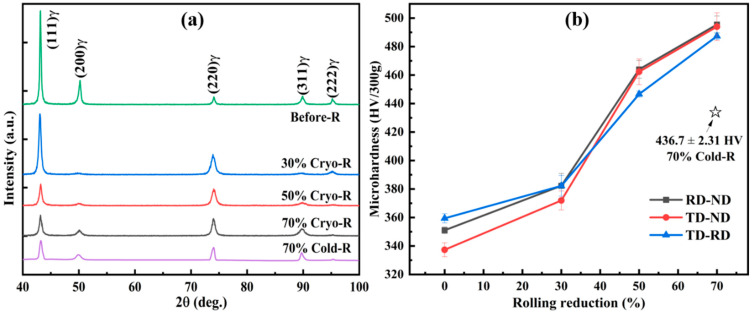
(**a**) The XRD patterns and (**b**) Vickers hardness values of SASS before and after Cryo/Cold-R with different reductions.

Figure 1 shows the evolution of the XRD patterns and Vicker hardness values of SASS before and after Cryo/Cold-R with different reductions. The diffraction peaks of all samples showed a single austenitic phase structure, which possesses a strong mechanical stability even at cryogenics. However, the DIM was observed in the Cryo-R samples by TEM analysis later; this inaccuracy was mainly due to its low volume fraction and nano-lath size. Moreover, the hardness value showed a monotonous increasing tendency with the cryogenic reduction increasing. However, it increased slowly during the early rolling process but showed a high increasing rate after the 30% reduction, which demonstrated the change in deformation modes. In addition, the hardness of the Cryo-R sample was obviously higher than that of the Cold-R sample at an equivalent strain of 70%, indicating that the former possessed a higher deformation stored energy due to the inhibition of dislocation recovery.

**Figure 2:**
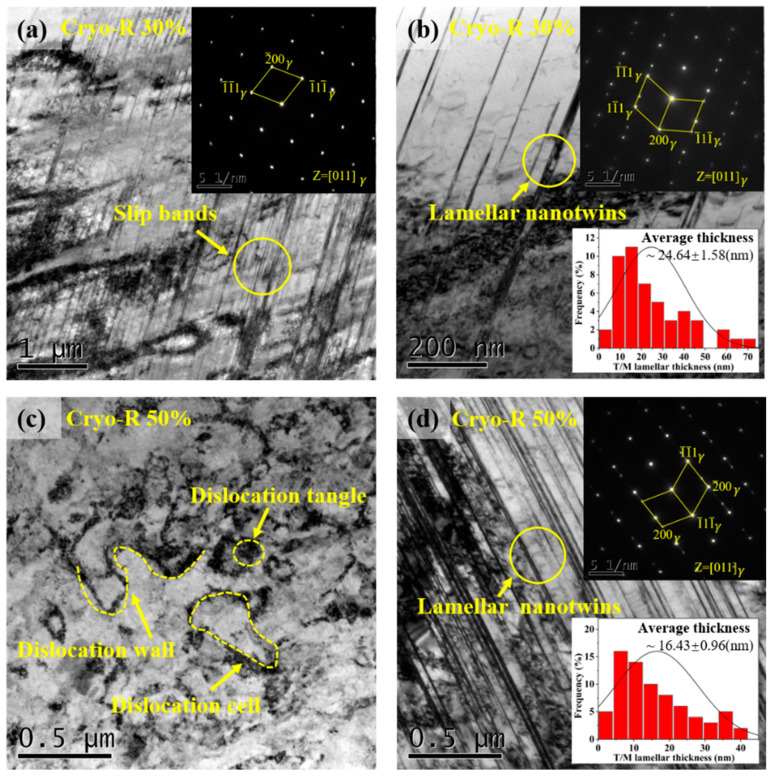
TEM micrographs of the SASSs after Cryo-R: (**a**) bright field image (BF) showing slip bands with 30% reduction; (**b**) BF showing lamellar NTs with 30% reduction; (**c**) BF showing dislocation structural with 50% reduction; (**d**) BF showing the lamellar NTs with 50% reduction.

Figure 2 depicts the typical TEM images of the Cryo-R SASS sample after 30% and 50% reductions. At low strains, a large number of slip bands were accompanied with dislocation cells or walls, and the motion of dislocations continuously crossed the matrix [24]. The corresponding selected area electron diffraction spots (SAED) shown in Figure 2a illustrate a single austenitic structure in this area, indicating that the martensitic transformation hardly occurs in the early cryogenic deformation stage. Furthermore, several nanotwins (NTs) were also observed, which was confirmed by SAED in Figure 2b. At low temperatures, the motion of dislocations was suppressed and instead the formation of twins was strongly promoted due to a decreased SFE [25]. According to the statistics, the average thickness of T/M lamellae was ~24.64 nm. With strain progressing to 50%, the further evolution of dislocation walls and cells not only inhibited dislocation slip, but also promoted an effective grain refinement [26]. More importantly, the enhanced nucleation driving force led to a significantly increased number density of NTs (Figure 2d), and the average size of T/M lamellar thickness was reduced to ~16.43 nm.

**Figure 3:**
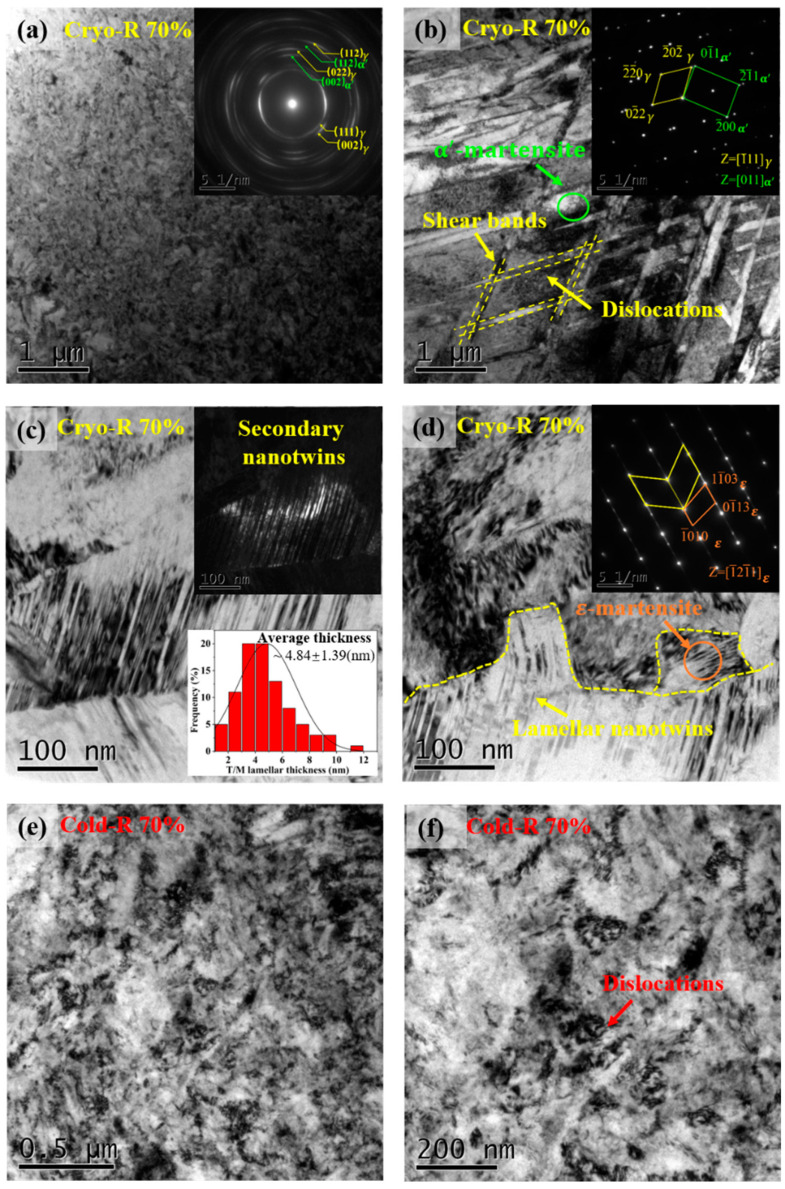
TEM micrographs of Cryo/Cold-R sample with 70% reduction: (**a**) BF and corresponding SAED; (**b**) BF showing the shear bands, α′ martensite; (**c**) BF showing the lamellar NTs, *ε*-martensite; (**d**) TEM micrograph of Cold-R sample with 70% reduction; (**e**) BF showing the matrix; (**f**) BF showing the dislocations.

When the rolling reduction increased to 70%, the austenitic grains were strongly refined; the ring-shaped SAED (Figure 3a) also indicated that the number of high-angle grain boundaries [27] obviously increased with a high degree of lattice distortion [28]. In addition, some special parallelogram regions of shear band interactions could be observed, and their interactions acted as the nucleation sites of the deformation-induced α′ martensite, which possessed a block morphology with a grain size of several micro-meters.

Moreover, some high-density secondary NTs could be observed between slip bands, and they showed a very thin T/M lamellar thickness of ~4.84 nm. The generation of secondary NTs was mainly caused by the strain gradient between adjacent grain boundaries [29]. Simultaneously, some nano-lath *ε* martensite also formed in the deformed ultra-fine grains (Figure 3c), which might be attributed to a limited dislocation storage and motion ability. In contrast, the formation of NTs or martensite was hardly observed in the Cold-R samples and only a large number of dislocation structures was evident in the grain interior.

As illustrated above, a hierarchical multiple deformed substructure, combined with dislocation cells, NTs, *ε* martensite and α′ martensite, could be found in the SASS during the cryogenic rolling process. The dislocation cells and walls dominate at low strains and the enhanced planar slip leads to the formation of slip bands. With the deformation progressing to a medium stage, a limited dislocation motion ability and increased flow stress result in the occurrence of high-density deformation twins and secondary slip bands, contributing to an obvious grain-refinement effect. Furthermore, the strain-induced α′ martensite nucleates in the interactions of shear bands and some stress-assisted *ε* martensite forms within the dislocation cell boundaries at large strains. The generation of such a multi-stage deformation mechanism is closely related to the temperature-controlled SFE and the conditions for the twin and martensite formation. Firstly, using the previously reported thermodynamic model by Saeed-Akbari et al. [30], and considering the change in SFE Γ by temperature besides alloy composition and grain size, Equation (1) is expressed as follows:

(1) $$\Gamma =2\rho {∆G}^{\gamma \to \varepsilon }+2\sigma^{\gamma /\varepsilon }+2\rho {∆G}_{ex}$$

where ρ is the molar surface density along $\left\{111\right\}$ planes and ${∆G}^{\gamma \to \varepsilon }$ and $\sigma^{\gamma /\varepsilon }$ are the free energy change and interfacial energy; ${∆G}_{ex}$ is the excess free energy due to the grain-size effect. Thus, the SFE values of the studied SASS are calculated as ~49.2 mJ·m^−2^ and ~22.1 mJ·m^−2^ at 293 K and 77 K, respectively. Moreover, according to the classic dislocation theory, the critical shear stress (CRSS) for the formation of the deformation twin can be expressed as follows [31]:

(2) $$\tau_{twin}=\frac{2\Gamma }{b_{p}}$$

where Γ is the SFE value, and $b_{p}$ is the Burgers vector of partial dislocations. Also, the driving force for the martensitic formation can be expressed as follows:

(3) $$-{∆G}^{\gamma \to \varepsilon }+\tau_{MT}sV_{m}=\int_{\gamma }^{\varepsilon }TdS$$

where $\tau_{MT}$ is the CRSS for the onset of *γ* → *ε* transformation, s is the homogeneous transformation shear strain, $V_{m}$ is the molar volume of austenite, $\int_{\gamma }^{\varepsilon }TdS$ is the change in entropy during *γ* → *ε* transformation. The parameter details are referred to in the previous work [32]. Thus, the CRSS values of twinning and martensite can be calculated as ~671 MPa and ~630 MPa at 293 K, and ~301 MPa and ~350 MPa at 77 K, respectively; that is, a lower CRSS results in the sequence of nanotwins and martensite when deformed at cryogenics, while they hardly form at RT due to the high CRSS values.

**Figure 4:**
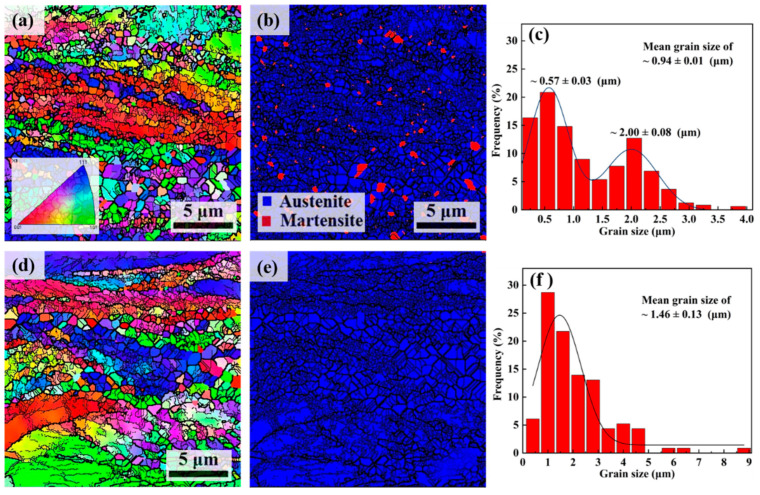
IPF maps, phase distribution maps and grain-size distribution of annealed SASS after (**a**–**c**) Cryo-R and (**d**–**f**) Cold-R.

Figure 4 shows the microstructural characteristic of annealed SASS after Cryo-R and Cold-R, including the inverse pole figures (IPFs), phase distribution maps and grain-size distributions from EBSD. A partial recrystallization process exists in both samples due to an insufficient annealing time. The annealed Cryo-R samples show a complex phase constitution of retained martensite and recrystallized and reversed austenite, which possesses a bimodal grain-size distribution, that is, ultrafine grains (~0.57 μm) and coarse grains (~2 μm). The reverse transformation of strain-induced martensite cannot be completed due to a low migration distance of *γ*/α′ phase boundary during the short-term annealing at 700 °C. However, the annealed Cold-R samples show a fully austenitic microstructure, consisting of coarse recovered grains and fine recrystallized grains, and their average grain size is higher than that of annealed Cryo-R samples. Compared with the Cold-R samples, the annealed Cryo-R samples show a larger heterogeneity and wider grain-size distributions; this phenomenon can be attributed to the hierarchical multiple precursors which experience competition among reverse transformation, recrystallization and recovery. For instance, the reversed austenite is prone to inherit the nano-lath morphology of martensite [33], while the growth of recrystallized grains in the deformation twin is strongly suppressed by the twin plane [34], and they exhibit a significantly smaller grain size than those nucleating in the dislocation cell boundaries. Moreover, the Cryo-R process leads to higher deformation stored energy than the Cold-R at an equivalent strain [35], which facilitates the improvement in the nucleation rate and contributes to a smaller average grain size [29].

**Figure 5:**
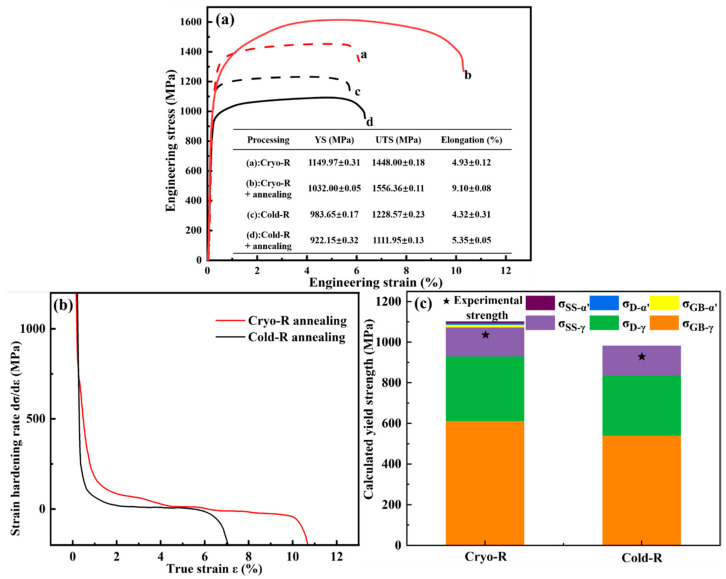
(**a**) Engineering stress–strain curves of the rolled and annealed SASS with Cryo-R and Cold-R processes (their tensile properties are shown in the inset table); (**b**) strain-hardening rate curve of Cryo-R/Cold-R annealing specimens; (**c**) calculated results of different strengthening mechanisms.

Figure 5 shows the engineering stress–strain and corresponding strain-hardening curves of the rolled and annealed SASS specimens. For the rolled specimens (a and c), the Cryo-R samples show higher YS and ultimate tensile strength (UTS) values but similar total elongation (TEL) compared with those of the Cold-R samples, which originate from a higher dislocation density (see the Supplementary Material) due to the inhibition of dislocation recovery at cryogenics. Moreover, the annealed Cold-R samples show an improved TEL value under the sacrifice of tensile strength compared with the rolled state, that is, the strength–ductility trade-off. However, the annealed Cryo-R sample shows simultaneously higher UTS and TEL values (~1556.36 MPa, ~9.1%) than those of the rolled state (~1448 MPa, ~4.93%), and the YS only decreases a little. Moreover, although the strain-hardening (SH) rate showed a decreasing tendency with deformation progressing, the SH value of the annealed Cryo-R sample was significantly higher than that of the annealed Cold-R sample during the whole tensile stage. As shown in Figure 5c, the superior strength–ductility synergy of the annealed Cryo-R sample is mainly attributed to multiple strengthening mechanisms [36,37], including the grain boundary strengthening, dislocation strengthening, and solid solution strengthening. The detailed calculations can be seen in the Supplementary Materials. Moreover, a significant hetero-deformation-induced hardening exists among the heterogeneous domains, contributing to the additional strain-hardening ability for the improvement of strain hardening and tensile ductility. In contrast, the annealed Cold-R samples show small microstructural heterogeneity and a large average grain size, thus exhibiting limited strengthening modes and unattractive strength–ductility combinations.

## 4. Conclusions

In summary, a good combination of strength and ductility was achieved in SASS by Cryo-R and subsequent annealing. The Cryo-R process resulted in the formation of a hierarchical multiple deformed substructure, including the dislocation cells, nanotwins and deformation-induced martensite, due to a low SFE and inhibited dislocation recovery. After annealing, the multiple precursors play an important role in obtaining the final heterogeneous structure, overcoming the strength–ductility trade-off. The high YS was mainly attributed to the ultra-fine microstructure, dislocation strengthening and retained martensite. Simultaneously, the hetero-deformation-induced hardening led to the improvement in strain hardening and tensile ductility.

## Data Availability

The data are included in the text.

## Supplementary Materials

The following supporting information can be downloaded at: https://www.mdpi.com/article/10.3390/ma16186298/s1.
