# Supplementary material for: Hierarchical Multiple Precursors Induced Heterogeneous Structures in Super Austenitic Stainless Steels by Cryogenic Rolling and Annealing
Source: Materials (Basel). 2023 Sep 20;16(18):6298. doi: 10.3390/ma16186298 (PMC10532896; doi:10.3390/ma16186298)
Supplement: Supplementary file 1 [file materials-16-06298-s001.zip › materials-2592256-supplementary.pdf]

Supplementary Material

Hierarchical multiple precursors induced heterogeneous structures in super austenitic stainless steels by cryogenic rolling and annealing

Duo Tan 1,*, Bin Fu 1,*, Wei Guan 1, Yu Li 1,*, Yanhui Guo 1, Liqun Wei 1 and Yi Ding 2

Strengthening mechanisms

As reported, the strengthening contributions of the multiphase (σ_T) are simply assumed according to the mixture rule [1] as follows:

$$\sigma_T = V(\gamma)\sigma(\gamma) + V(\alpha')\sigma(\alpha') \quad (1)$$

where $V(\gamma)$ and $V(\alpha')$ are the volume fractions of austenite (~97.3%) and martensite (~2.7%), respectively, $\sigma(\gamma)$ and $\sigma(\alpha')$ are the yield strength values of austenite and martensite, respectively. The strengthening mechanisms of constituents can be summarized into three categories: grain-boundary strengthening, dislocation strengthening, solid solution strengthening and precipitation strengthening. The precipitation is difficult under the condition of low annealing temperature and short annealing time, and the precipitates were hardly found in the experimental TEM characterization, so the precipitation strengthening can be ignored in this work. Thus, the strengthening contributions of individual mechanisms in the annealed Cold-R and Cryo-R samples can be illustrated as follows:

$$\sigma_s = \sigma_{GB} + \sigma_D + \sigma_{SS} \quad (2)$$

where σ_s represents the yield strength; σ_{GB}, σ_D and σ_{SS} represent the contribution of grain-boundary strengthening, dislocation strengthening and solid solution strengthening, respectively.

1. Grain-boundary (GB) strengthening (Hall–Petch effect)

The GB strengthening mechanism is usually described by the Hall–Petch equation[2]:

$$\sigma_{GB} = \sigma_0 + K_{HP} d_m^{-0.5} \quad (3)$$

where the σ_0 values are 180 and 120 MPa for austenite and martensite [3], respectively. The coefficient K_{HP} values of austenite and martensite are 435 and 210 MPa·μm$^{-1/2}$ [4], respectively. From the EBSD statistics (Fig. 4c and 4f), the average grain size d_m values of the austenite and martensite in the annealed Cryo-R sample were ~0.94 and ~0.31 μm, respectively, and the d_m value

of the austenite in the annealed Cold-R sample was ~1.46 μm. Thus, the contribution to GB strengthening in both samples was shown in Tab. S1:

Table S1. GB strengthening contribution of γ/α' in the annealed Cryo-R and Cold-R samples.

GB strengthening	Annealed Cryo-R	Annealed Cold-R
$\sigma_{GB-\gamma}$ (MPa)	611.6	540
$\sigma_{GB-\alpha'}$ (MPa)	13.4	/
σ_{GB} (MPa)	625	540

2. Dislocation strengthening

To evaluate and compare the role of the residual dislocations to strengthening in SASSs, the Bailey–Hirsch relationship [5] was applied in the current study:

$$\sigma_D = \alpha M G b \rho^{0.5} \quad (4)$$

where M is the mean orientation factor, α is the constant, G is the shear modulus, b is the burgers vector. The values of detailed parameters were shown in table S2.

Table S2 Physical quantities for strengthening mechanism calculations.

Symbol	Meaning	Values	Unit	References
b	Burgers vector	0.286	nm	[2]
M	Mean orientation factor	3.06	Dimensionless	[6, 7]
G	Shear modulus	82	GPa	[8]
α	Constant	0.3	Dimensionless	[9]

The dislocation density ρ can be calculated by the following formula [10] from the KAM:

$$\rho = 2\theta / b\Delta x \quad (5)$$

where θ is the average KAM value of the selected zone, as shown in Fig. S1. The θ values of austenite and martensite in the annealed Cryo-R samples are 1.65 and 2.08, respectively, and the θ value of austenite in the annealed Cold-R sample is 1.35. Δx is the step size (~0.05 μm). Thus, the as-calculated dislocation density and the related dislocation strengthening contributions are shown in Tab. S3.

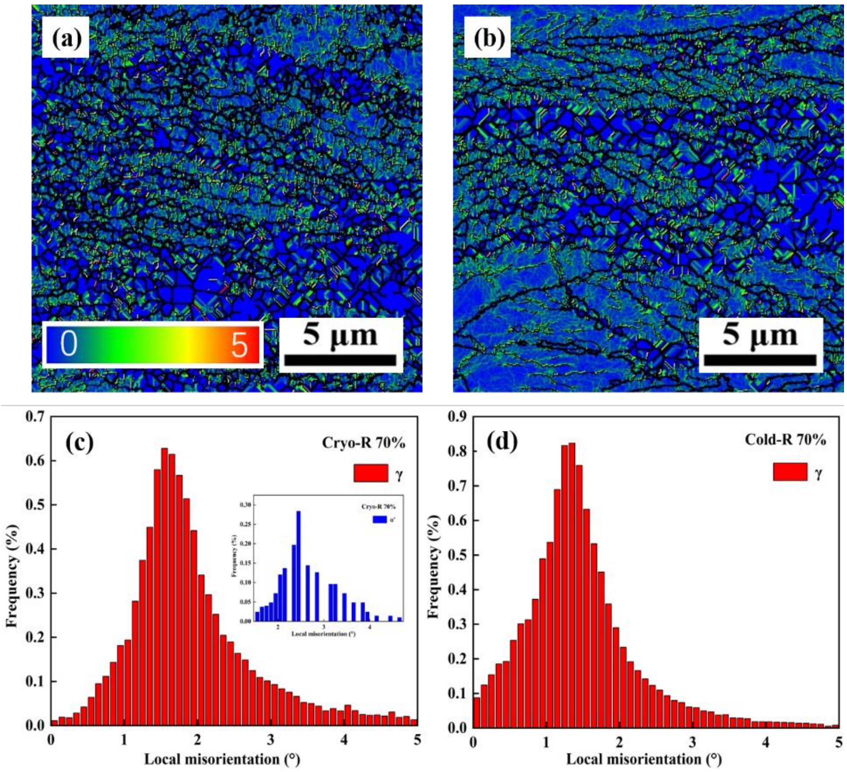

Fig S1. (a, b) The KAM distributions and (c, d) average KAM values of the constituent phases in the annealed Cryo-R, Cold-R samples.

Table S3. Dislocation strengthening contribution of the annealed Cryo-R and Cold-R samples

Dislocation strengthening	Annealed Cryo-R	Annealed Cold-R
ρ_{γ}	$2.3*10^{14}$	$1.9*10^{14}$
$\rho_{\alpha'}$	$2.9*10^{14}$	/
$\sigma_{GB-\gamma}$ (MPa)	318.2	540
$\sigma_{GB-\alpha'}$ (MPa)	9.91	/
σ_{GB} (MPa)	625	540

3. Solid solution strengthening

The solid solution strengthening (σ_{ss}) can be expressed by the equation [11, 12]:

$$\sigma_{ss-a} = 68 + 20 \times (\%Si) + 3.7 \times (\%Cr) + 354 \times (\%C) \quad (6)$$

$$\sigma_{ss-m} = 77 + 32 \times (\%Mn) + 83 \times (\%Si) - 3.1(\%Cr) + 5000 \times (\%C) \quad (7)$$

the chemical composition of SASS was listed as follows: 0.024 C, 0.38 Si, 0.98 Mn, 0.027 P, 0.004 S, 16.9 Cr, 13.23 Ni, 5.3 Mo, 0.13 N and balanced Fe, wt. %. Thus, the solid solution strengthening of the annealed Cryo-R and Cold-R samples are shown in Tab. S4.

Table S4 Solid-solution strengthening contribution of the annealed Cryo-R and Cold-R samples

Solid solution strengthening	Annealed Cryo-R	Annealed Cold-R
$\sigma_{ss-\gamma}$	142.6	146.6
$\sigma_{ss-\alpha'}$	5.6	/
σ_{ss} (MPa)	148.2	146.6

As illustrated above, the contributions of individual strengthening mechanisms to the YS can be summarized as Tab. S5. The total strengthening contributions of the annealed Cold-R and Cryo-R samples are calculated as ~1101.3 and ~981.8 MPa, corresponding well to the experimental YS results. Moreover, the total strength contributions of the martensitic phase are calculated as ~30 MPa, which contributes little to the overall YS. However, the existence of retained martensite may result in strong stress partitioning [13] between constituent phases, thus influencing the mechanical stability of austenite and related transformation-induced plasticity (TRIP) effect.

Table S5. Strengthening contributions of individual mechanisms in the annealed Cryo-R and Cold-R samples.

Strengthening contributions	σ_{GB} (MPa)	σ_D (MPa)	σ_{SS} (MPa)	YS $_{Calculation}$ (MPa)	YS $_{Experiment}$ (MPa)
Annealed Cryo-R	625	328.1	148.2	1101.3	1032

Annealed<br>Cold-R	540.0	295.2	146.6	981.8	922.1

SFE calculation

According to the previous models, the SFE can be calculated by the following formula [14]:

$$\Gamma = 2\rho_m \Delta G^{\gamma\to\varepsilon} + 2\sigma^{\gamma/\varepsilon} + 2\rho\Delta G_{ex} \quad (8)$$

where ρ_m is the molar surface density along $\{111\}$ planes;

$$\rho_m = \frac{4}{\sqrt{3}a^2 N} \quad (9)$$

where a is the lattice constant ~0.361 nm, N is Avogadro's constant, $\sigma^{\gamma/\varepsilon}$ is the interfacial energy ~8 mJ/m^2 [15], and ΔG_{ex} is the excess free energy due to the grain size effect.

$$\Delta G_{ex} = 170.06\exp\left(\frac{-d_m}{18.55}\right) \quad (10)$$

From the previous thermodynamic simulations, the free energy change $\Delta G^{\gamma\to\varepsilon}$ can be calculated by using the model as follows[16, 17]:

$$\Delta G^{\gamma\to\varepsilon} = \sum_i \chi_i \Delta G_{\mathrm{i}}^{\gamma\to\varepsilon} + \sum_{ij} \chi_i \chi_j \Omega_{ij}^{\gamma\to\varepsilon} + \Delta G_{\mathrm{mg}}^{\gamma\to\varepsilon} \quad (11)$$

where χ_i is the molar fraction of the alloy element i, $\Delta G_{\mathrm{i}}^{\gamma\to\varepsilon}$ is the change in the Gibbs energy of each element i upon $\gamma \to \varepsilon$ phase transformation, the excess free energy $\Omega_{ij}^{\gamma\to\varepsilon}$ is the first-order interactions between element i and j, $\Delta G_{\mathrm{mg}}^{\gamma\to\varepsilon}$ is the magnetic contribution due to the paramagnetic-to-antiferromagnetic transition [17]. Some parameters of $\Delta G_{\mathrm{i}}^{\gamma\to\varepsilon}$ and $\Omega_{ij}^{\gamma\to\varepsilon}$ are given in Tab. S6.

Table S6. Thermodynamic functions of some parameters to calculate $\Delta G^{\gamma\to\varepsilon}$.

Parameter	Thermodynamic function (J/mol)
$\Delta G_{\mathrm{Fe}}^{\gamma\to\varepsilon}$	-1828.4+4.686T
$\Delta G_{\mathrm{Mn}}^{\gamma\to\varepsilon}$	3970-1.7T
$\Delta G_{\mathrm{Al}}^{\gamma\to\varepsilon}$	5481.04-1.799T
$\Delta G_{Si}^{\gamma\to\varepsilon}$	-560-8T
$\Delta G_{\mathrm{Cr}}^{\gamma\to\varepsilon}$	-2846-0.163T
$\Delta G_{Mo}^{\gamma\to\varepsilon}$	-3650-0.63T
$\Delta G_{Ni}^{\gamma\to\varepsilon}$	1046+1.255T
$\Delta\Omega_{\mathrm{FeMn}}^{\gamma\to\varepsilon}$	-9135.5+15282.1χ_{Mn}

$\Delta\Omega^{\gamma\to\varepsilon}_{\mathrm{FeAl}}$	3323
$\Delta\Omega^{\gamma\to\varepsilon}_{\mathrm{FeCr}}$	2095
$\Delta\Omega^{\gamma\to\varepsilon}_{FeNi}$	2095
$\Delta\Omega^{\gamma\to\varepsilon}_{\mathrm{CrNi}}$	4190
$\Delta\Omega^{\gamma\to\varepsilon}_{\mathrm{FeSi}}$	285+3250 $(\chi_{Fe} - \chi_{Si})$

Here T is the temperature in Kelvin, and $\Delta G_i^{\gamma\to\varepsilon}$ and $\Delta\Omega_{ij}^{\gamma\to\varepsilon}$ are in J/mol. According to the chemical composition and deformation temperature, the SFE values of the studied SASS are calculated as ~49.2 mJ·m^{-2} and ~22.1 mJ·m^{-2} at 293 K and 77 K, respectively.

References:

[1] R. Zhu, Microstructure design of low alloy transformation-induced plasticity assisted steels, Dissertations & Theses - Gradworks (2013).

[2] K. Ma, H. Wen, T. Hu, T.D. Topping, D. Isheim, D.N. Seidman, E.J. Lavernia, J.M. Schoenung, Mechanical behavior and strengthening mechanisms in ultrafine grain precipitation-strengthened aluminum alloy, Acta Materialia 62 (2014) 141-155.

[3] A. Belyakov, M. Odnobokova, I. Shakhova, R. Kaibyshev, Regularities of Microstructure Evolution and Strengthening Mechanisms of Austenitic Stainless Steels Subjected to Large Strain Cold Working, Materials Science Forum 879 (2016) 224-229.

[4] M. Park, M.S. Kang, G.-W. Park, H.C. Kim, H.-S. Moon, B. Kim, J.B. Jeon, H. Kim, H.-S. Park, S.-H. Kwon, B.J. Kim, Effects of Annealing Treatment on the Anisotropy Behavior of Cold-Rolled High-Manganese Austenite Stainless Steels, Metals and Materials International 27(10) (2020) 3839-3855.

[5] H. Wen, T.D. Topping, D. Isheim, D.N. Seidman, E.J. Lavernia, Strengthening mechanisms in a high-strength bulk nanostructured Cu–Zn–Al alloy processed via cryomilling and spark plasma sintering, Acta Materialia 61(8) (2013) 2769-2782.

[6] Z. Li, L. Fu, J. Peng, H. Zheng, A. Shan, Effect of annealing on microstructure and mechanical properties of an ultrafine-structured Al-containing FeCoCrNiMn high-entropy alloy produced by severe cold rolling, Materials Science and Engineering: A 786 (2020).

[7] T.R. Smith, J.D. Sugar, C. San Marchi, J.M. Schoenung, Strengthening mechanisms in directed energy deposited austenitic stainless steel, Acta Materialia 164 (2019) 728-740.

[8] M.M. Abramova, N.A. Enikeev, R.Z. Valiev, A. Etienne, B. Radiguet, Y. Ivanisenko, X. Sauvage, Grain boundary segregation induced strengthening of an ultrafine-grained austenitic stainless steel, Materials Letters 136 (2014) 349-352.

[9] J. Peng, Z. Li, L. Fu, X. Ji, Z. Pang, A. Shan, Carbide precipitation strengthening in fine-grained carbon-doped FeCoCrNiMn high entropy alloy, Journal of Alloys and Compounds 803 (2019) 491-498.

[10] Y. Guo, S. Zhang, J. Chen, B. Fu, Z. Wang, L. Pang, L. Wei, Y. Li, Y. Ding, The contribution of retained martensite to the high yield strength and sustainable strain hardening of a

hierarchical metastable austenitic stainless steel, Materials Science and Engineering: A 866 (2023).
[11] I. Gutiérrez, M.A. Altuna, Work-hardening of ferrite and microstructure-based modelling of its mechanical behaviour under tension, Acta Materialia 56(17) (2008) 4682-4690.
[12] A. Perlade, O. Bouaziz, Q. Furnémont, A physically based model for TRIP-aided carbon steels behaviour, Materials Science and Engineering: A 356(1-2) (2003) 145-152.
[13] C. Song, H. Yu, J. Lu, T. Zhou, S. Yang, Stress partitioning among ferrite, martensite and retained austenite of a TRIP-assisted multiphase steel: An in-situ high-energy X-ray diffraction study, Materials Science and Engineering: A 726 (2018) 1-9.
[14] T.L. Achmad, W. Fu, H. Chen, C. Zhang, Z.-G. Yang, Effects of alloying elements concentrations and temperatures on the stacking fault energies of Co-based alloys by computational thermodynamic approach and first-principles calculations, Journal of Alloys and Compounds 694 (2017) 1265-1279.
[15] E.I. Galindo-Nava, P.E.J. Rivera-Díaz-del-Castillo, Understanding martensite and twin formation in austenitic steels: A model describing TRIP and TWIP effects, Acta Materialia 128 (2017) 120-134.
[16] A. Saeed-Akbari, J. Imlau, U. Prahl, W. Bleck, Derivation and Variation in Composition-Dependent Stacking Fault Energy Maps Based on Subregular Solution Model in High-Manganese Steels, Metallurgical and Materials Transactions A 40(13) (2009) 3076-3090.
[17] S. Curtze, V.T. Kuokkala, A. Oikari, J. Talonen, H. Hänninen, Thermodynamic modeling of the stacking fault energy of austenitic steels, Acta Materialia 59(3) (2011) 1068-1076.
